# Occupational Physicians’ Reasoning about Recommending Early Return to Work with Work Modifications

**DOI:** 10.1371/journal.pone.0158588

**Published:** 2016-07-01

**Authors:** Ritva Horppu, Kari-Pekka Martimo, Eira Viikari-Juntura, Tea Lallukka, Ellen MacEachen

**Affiliations:** 1 Prevention of Work Disability, Finnish Institute of Occupational Health, Helsinki, Finland; 2 School of Public Health and Health Systems, University of Waterloo, Waterloo, Canada; 3 Centre for Research on Work Disability Policy, Institute for Work & Health, Toronto, Canada; University of Perugia, ITALY

## Abstract

Previous research indicates that work modifications can effectively enhance return to work (RTW) at an early stage of work disability. We aimed to examine how occupational physicians (OPs) reason about recommending early return to work (RTW) with work modifications. Pre-defined propositions regarding the use of work modifications in promoting early RTW were discussed in four focus groups with altogether 11 Finnish OPs. Discussions were audio recorded, and the transcribed data were analyzed using qualitative content analysis. Five different rationales for supporting early RTW were identified: to manage medical conditions, to enhance employee well-being, to help workplace stakeholders, to reduce costs to society, and to enhance OP’s own professional fulfillment. However, OPs identified situations and conditions in which early RTW may not be suitable. In addition, there were differences between the OPs in the interpretation of the rationales, suggesting variation in clinical practice. In conclusion, encouraging early RTW with work modifications was perceived by OPs as a meaningful task and, to a large extent, beneficial for employees and several stakeholders. However, this practice was not accepted without consideration to the RTW situation and context. If early RTW and work modifications are to be promoted, OPs should be offered education that addresses their views regarding this practice.

## Introduction

The likelihood of return to work (RTW) decreases rapidly with increasing duration of sickness absence [1], therefore, prevention of unnecessary absence from work should always be a priority. Work modifications are often necessary to enable RTW with reduced work ability. Interventions at different stages of disability indicate that low cost and relatively quickly administered interventions—such as work modifications—can be cost-effective at an early stage (< 6 weeks) of work disability [1]. Randomized trials have shown promising results, especially with respect to musculoskeletal disorders [2–5], while the optimal timing and content of interventions for mental disorders are less clear [3, 6–7].

Legislation has been changed in many jurisdictions, e.g., UK and Norway, regarding work modifications to enable work retention instead of sickness absence [8–9]. However, physicians may have little knowledge of their patients’ work or their contacts to the workplaces may be limited, impeding negotiations about necessary work modifications [10–11].

In Finland, all employers must provide occupational health services (OHS) to their employees, purchasing the services from outside or arranging them as in-house services. Occupational physicians (OPs) provide general practitioner -level medical care in addition to the statutory preventive OHS such as work-related health surveillance. Employees may self-refer to an OP even at an early stage of any disabling medical problem. Because statutory OHS include regular assessments of health and safety in the workplace, familiarity with the employees’ work environment creates an ideal opportunity to encourage early RTW by means of accommodated work. Although both the legislation on OHS and the training of OPs support this approach, early RTW is not a standard practice. Suggesting work modifications to the employee and advising the supervisor either by phone or writing a recommendation relies mostly on the consideration and motivation of OPs. This analysis is derived from a larger study designed to activate Finnish OPs to engage in early RTW practices [12].

The study is informed by theoretical frameworks for understanding practitioners’ behavior change [13–15]. Adoption of new practices may be impeded or facilitated by practitioners’ knowledge of and reasoning about such practices. Previous studies have explored health care practitioners’ views about *when* and *how* to support early RTW, and their experiences of the barriers and facilitators of successful RTW [16–23]. However, these studies have not investigated directly *why* they would or would not, in the first place, bring up this possibility with employees or employers.

The aim of this qualitative study was to examine how OPs reason about encouraging early RTW by means of work modifications, i.e., how they perceive the rationales for and meaning of this practice. We explored the variation of OPs’ reasoning, emerging from focus group discussions. More specifically, we aimed to identify what is central for OPs in relation to encouraging early RTW by studying both the content of the discussions as well as areas of agreement and disagreement between the participants.

## Methods

This study is part of an educational intervention project aimed at enhancing early RTW via OPs’ increased recommendations of work modifications among employees with musculoskeletal pain or depressive symptoms’ (ISRCTN74743666). Pilot interviews with four Finnish OPs showed that participants had varied rationales for engaging in early RTW. In order to plan adequate interventions to promote the initiation of work modifications, a more in-depth understanding of OPs’ reasoning of this practice is needed. Focus group discussions were considered an optimal approach for this study, because they encourage the participants to prompt each other, allowing a deeper insight into their reasoning [24–27].

### Participants and Procedure

A purposive sampling approach was used in order to compose focus groups in which participants are similar enough, but have sufficient variability in order to allow for differences in experiences and perceptions [28–29]. We aimed to recruit participants of both genders, and of varying years of experience in OHS.

Ten OPs specializing in occupational diseases at the Finnish Institute of Occupational Health by the time of the study were asked to participate. Twenty OPs were contacted after they had attended a further education course related to mentoring junior OPs. They were all approached by e-mail by the first author. Six OPs in each group agreed to participate. Time constraints were the main reason for non-participation.

Altogether 11 OPs (7 women and 4 men) met in four focus groups, and one of the intended participants was unable to attend. Two were all-female groups and two groups comprised both genders. Three groups included three participants, and one group discussion was held with two participants. The final focus group size would have been larger if all committed participants had been able to attend; however, circumstances intervened.

The median of the participants’ job tenures in various types of OHS was 14 years (range 4 to 37 years). One participant had worked in OHS less than 5 years, three participants had 5–10 years of experience in OHS, four participants had worked in OHS 11–20 years, and three participants had more than 20 years of experience in OHS.

The small groups ultimately allowed for in-depth discussion on the topic. We wanted to hear physicians explain and justify their RTW reasoning to each other. All participants were familiar with supporting RTW, and they described authentic cases which they had been involved with personally. Focus group discussions took place at the Finnish Institute of Occupational Health in Helsinki, and they lasted approximately 90 minutes. Discussions were audio recorded and transcribed verbatim. Field notes were written after every focus group in order to capture data on group interaction.

In order to challenge the participants to deliberate thoroughly and to express divergent arguments about encouraging early RTW with accommodated work, we used pre-defined propositions instead of ordinary open-ended questions. We produced five propositions on this topic on the basis of knowledge provided in scientific literature and the pilot interviews:

1. To continue at work or to return to work early using work modifications is often more beneficial than sickness absence.
2. Temporary work modifications are useful in both musculoskeletal and mental disorders.
3. It is the OP’s responsibility to initiate work modifications.
4. The supervisor’s willingness to implement work modifications depends on the personal characteristics of the worker with disability.
5. Confidentiality of health-related issues makes the use of work modifications difficult at workplaces.

The first author served as a moderator in all focus groups. In the beginning of the discussions general information on focus group discussion was given, and the moderator emphasized that different points of views were appreciated. To begin with, participants were asked to describe a case where they had been engaged in early RTW. After presenting a proposition the moderator asked participants to state whether they agreed or disagreed with it and to provide their reasoning for their views. Subsequent questions were used to follow up the arguments or accounts of experiences. The moderator actively sought to elicit contrasting perspectives to explore the rationales more in-depth. No suggestions were made to reach a consensus in the discussions. In the end of each group discussion, participants were asked to raise other issues related to early RTW that they considered important but which had not been discussed so far.

### Analysis

Qualitative content analysis [28–32] led to an analytic focus on physician’s reasoning for engaging or not engaging in supporting early RTW and their reasoning for applying or not applying accommodated work to ensure early RTW. Transcripts from the first three focus groups were read several times. Using a constant comparative method, preliminary categories were defined for coding the data. Initial categories focused on what was aimed at by recommending early RTW, e.g., recovery from a disease, benefiting the stakeholders, or financial benefits. Further, subcategories were distinguished according to who would benefit from applying or not applying this practice, i.e., individual employees, companies, supervisors, co-workers, society, or the OPs themselves. Finally, five main categories (i.e., the reasons for supporting and not supporting early RTW) and related sub-categories were developed and systematically applied to the data of the first three focus groups. The analysis proceeded by looking for similarities and differences within groups and between groups.

Preliminary explanations to account for the identified similarities and differences were developed. Deviant cases were sought and examined. Emerging explanations were revised in the light of disconfirming data. Finally, a fourth focus group discussion was convened. The analysis scheme was applied to the new data. Emerging explanations were discussed by all authors together and were found to fit, with no new categories or explanations needed. The multi-disciplinary research team included specialists from different fields (occupational medicine, rehabilitation medicine, public health, sociology, social psychology, and education).

### Ethical Considerations

The study was accepted by the Coordinating Ethics Committee of Helsinki and Uusimaa Hospital District (approval number 35/13/03/00/2013). The interviews of this study were mentioned as one sub-study of the larger intervention study (ISRCTN74743666), and the information leaflet for the participants regarding the interviews and the informed consent form were included in the application and accepted by the Ethics Committee. The participants were provided with due information about the research, and they were told that all collected data would be confidential and anonymous. Written consent was obtained from the participants for the recording and transcriptions of the discussions. The participants were informed that they were entitled to request any of their comments to be erased from the transcripts later if they decided so. Nobody made this kind of request. Confidentiality has been kept in the analytical and presentational practices by using pseudonyms of the participants.

## Results

The results focus on physician’s reasons for supporting or not supporting early RTW before full recovery, a relatively new concept and practice in workplaces in Finland. Five different reasons for supporting and not supporting early RTW are presented: to manage medical conditions, to enhance employee well-being, to help workplace stakeholders, to reduce costs to society, and to enhance OP’s own professional fulfillment.

### 1. Early RTW to Manage Medical Conditions

The focus of the first area of reasoning was early RTW as a management of medical conditions. RTW can be perceived as a proactive treatment for a medical condition, e.g. minor depressive symptoms or musculoskeletal pain. Temporary work modifications can be used to decrease the physical or psychological exposures that are considered to have triggered or worsened the symptoms. In this way modified work can be a sufficient cure without a need for additional treatment. In addition, when work modifications were not considered to enhance recovery directly, they were seen to provide safe working conditions during recovery, medical treatment or waiting period for examinations or treatments.

Using early RTW as a means of managing a medical condition was brought up spontaneously in all focus groups. However, some reservations were also expressed with regard to this rationale. OPs emphasized that RTW that is too early may set back recovery. The groups were unanimous that some conditions (e.g., acute psychosis or severe sciatica) are inherently unsuitable for early RTW. In these cases, either early RTW hinders recovery or there are no duties which the disabled employee could perform safely and productively.

In addition, participants had divergent views of the application of this rationale. There were negotiations within groups about whether there are some (minor) medical conditions that should prompt an OP avoid suggesting early RTW for an employee. For example, it was stated that employees with burnout should be categorically ruled out of the option of early RTW. In contrast, it was argued that OPs should avoid preconceived classifications of medical conditions unsuitable for early RTW.

*Then you’ve got this large group of burnout, depressed and perfectionist people or supervisors that you’ve just got to drive away from work, because they are unable to delimit their duties. … To me they appear to be a group of professionals to whom you simply state that ‘your keys, mobile phones and lap tops on the desk, please. Here is your sick note, and please close the door behind you’. Sometimes you have to be a little bit rude*.

*(Bertha,* *group 1)*

*You sure should consider if it would be possible to use these temporary work modifications − be it a question of mental disorders or musculoskeletal pain. In that sense I agree with the proposition. Otherwise it will go pretty much case by case. But you shouldn’t categorize that they only apply to this symptom but not at all to that one*.

*(Adam,* *group 1)*

There were also negotiations within groups about whether the causes of medical conditions determine if early RTW via work modifications is suitable.

*I think that in case of mental disorders, if one is exhausted and it’s clearly caused by the job, work modifications is the primary option. But if depression is caused by private matters, then it’s quite difficult to arrange modified work*.

*(Fanny,* *group 3)*

*But on the other hand, it may be the very work community that is exactly right cure. One may think that ‘it’s nice to be able to be at work instead of having to ponder on my private matters’*.

*(Hilda,* *group 3)*

In addition, it was negotiated whether OPs should recommend organizing accommodated work to ensure early RTW if being at work did not actively enhance recovery.

*Musculoskeletal pain is often rather short-term, so in that case temporary work modifications could be applied, or is it so? If we are dealing with a short-term worsening period, then it might be most reasonable and effective that one is totally absent from work*.

*(Bertha,* *group 1)*

*If the patient has such a condition that he/she could return to work earlier with temporary work modifications, then I think we have a statutory duty to try to find out what the situation is, for example, to give a call to the supervisor. I do not mean that we should push the patient to return to work nor to try to pressure in any way the supervisor to accept that, but to tell that this sort of work modification is possible. Surprisingly often people want to return to work but they really are concerned if they can manage*.

*(Carl,* *group 1)*

### 2. Early RTW to Enhance Employee Well-Being

The second area of reasoning focused on employees’ well-being, in contrast to the restricted focus on the medical conditions of the previous category. Work modifications were viewed as a means of supporting employees’ existing positive resources by creating a safe opportunity for motivated employees to stay at work. Participants described cases, in which employees either declined sick leave or requested termination of sick leave, because, for example, they felt frustrated at home.

In addition, early RTW was perceived as a way to prevent negative psychosocial consequences of prolonged sick leaves. These consequences were e.g., reduced work motivation, increased fears related to RTW, fear of being discharged from work, threatened position as an appreciated member of the work community, ending up being bullied because workmates had to do extra duties during a long sick leave, or not returning to work at all after a long sick leave.

This reasoning about enhancing employees’ well-being was brought up spontaneously in all focus groups. However, participants had divergent views of the application of this rationale to workers of all ages. Although reduced work motivation of workers was a common focus of worries, it was questioned whether OPs should be concerned about the work motivation of older employees.

*I sometimes stray into philosophical pondering, such as that if we are treating painful shoulder and a person says that ‘I sure have been working for such a long time that it would be time for the younger to take over’, then to me it’s a clear open message about the motivating factors*.

*(Bertha,* *group 1)*

### 3. Early RTW to Help Workplace Stakeholders

The third rationale considered the relationship between early RTW and workplace stakeholders. First, early RTW was perceived as producing financial benefits for companies. Shorter sick leaves will help companies to keep costs at a minimum. It was stated that via temporary work modifications the employers do not need to pay for sick leave days, and skillful employees are doing at least some type of productive work. It was also suggested that, in the long run, companies’ expenses for permanent disability are reduced when employees with disability are supported to stay at work.

This rationale was brought up spontaneously in three focus groups. However, participants remarked that certain types of companies, (e.g., small companies in the service sector, or companies doing piecework) may not benefit financially from encouraging early RTW via modified work. If a disabled employee is performing only a part of his/her normal service duties, fewer customers are being served or fewer goods are being produced.

Some participants questioned the credibility of the financial benefit for the company. They were not convinced that adequate evidence exists that early RTW will reduce company costs. They also pondered whether reduced company costs was an appropriate reason for early RTW practice.

*I also think about that trend, actually I haven’t thought about it earlier, but it comes to my mind now, that probably one thing are these private OH services that make contracts with companies. Costs need to be minimized and sick leaves are too long. And each time a company is choosing a new private OHS provider, the marketing has been such as ‘with us your sick leaves will fall or decrease’*.

*(Doris,* *group 2)*

*Controlling of sick leaves is pretty intense in some companies, and another thing is the cost of a sick leave day. And the economy of the state is what it is, and times are tough, and nobody should have days off now*…

*(Elsa,* *group 2)*

*The state of economy is poor, and with the private health care sector, and the production of research evidence may have been purpose-oriented, but it may well be credible*.

*(Doris,* *group 2)*

A second way early RTW was seen to affect workplace stakeholders was via the well-being of supervisors and/or coworkers. Supervisors benefit because they do not need to organize and advise substitute employees. Coworkers benefit because the disabled employees do at least some duties, instead of shifting additional work load to their colleagues. It was also suggested that work modifications enhance a sense of security in work communities. When employees notice that the employer is committed to supporting a colleague’s work career, they may be reassured that they, too, will be offered similar possibilities, if needed.

However, some participants expressed reservations for this rationale. It was argued that sometimes work modifications, e.g., part-time work, will rather increase than reduce coworkers’ workload. Therefore, temporary work modifications were considered as most suitable to workplaces with reciprocity between coworkers and trust in supervisors’ justice. In addition, it was suggested that in some workplaces accommodated work will not reduce but increase supervisors’ workload.

*A chief nurse said to me that ‘not another person with lowered work ability will be employed in my unit. I already have difficulties in putting together shift lists because these can’t take night shifts, and those are not capable of lifting, and this one has that limitation, and that one has this limitation. And then you’ve got these mothers of small children who don’t want to start their shift before 7 a.m. and need to go before 3 p.m’*.

*(Bertha,* *group 1)*

### 4. Early RTW to Reduce Costs to Society

The focus of the fourth rationale was on the benefits of this practice to society. Early RTW was perceived as a means of encouraging work participation. It was stated that all citizens benefit when as many of us as possible are working and paying taxes. Thus, early RTW should always be considered as the first option. In addition, early RTW was perceived as a means of reducing societal costs caused by work disability. These costs are mostly related to prolonged sick leaves, which may be prevented by work modifications.

This area of reasoning was brought up spontaneously in all groups, but reservations for the rationale were also expressed. It was suggested that although promoting early RTW may be beneficial for the society as a whole, it should not become a moral norm that oblige all individuals. For example, in case an employee returning to part-time work suffers financially compared to full-time sick leave, he/she should not be expected to sacrifice personal interests for the society’s good.

It was further argued that if OPs are expected to urge all eligible employees to choose early RTW via work modifications instead of full sick leave, some employees (e.g. persons having overly high respect for doctors, or timid persons) may consent to an OP’s suggestion even if they do not genuinely regard it as being in their best interest.

*There always is a possibility that an individual is mistreated, and people will be treated differently. Some people are more capable of holding their own and demanding. The doctor may become less neutral when evaluating ‘should I prescribe a full sick leave or a part-time sick leave?’ The employee of course doesn’t have to agree with the doctor’s suggestion, but the doctor can always also persuade: ‘This is a healthy and good alternative for you, and we’ll maintain your work ability’*.

*(Doris,* *group 2)*

### 5. Early RTW as a Means of Professional Fulfillment of the OPs

The focus of the fifth rationale was on the OP. Promoting early RTW was described as enhancing OPs’ professional fulfillment. It was suggested that supporting an employee’s work career in a long run by, e.g., the means of work modifications, is more meaningful than prescribing recurrent sick leaves. In addition, the types of tasks that OPs engage in when negotiating about work modifications with employees and employers were described as satisfying.

This area of reasoning was brought up spontaneously in three groups. However, limits of application were also expressed. Some duties related to work modifications were seen as satisfying while other duties were experienced as demanding and stressing.

*Employers are very determined… You may receive strong clear ultimatums from the employers about how issues must be handled. I personally feel more uncertain with employers, but it’s always somehow easier to communicate with the patient*.

*(Doris,* *group 2)*

This rationale was also questioned in another group. It was argued that professional fulfillment is not a decisive factor for engaging or not engaging in early RTW. The group members described this practice as often discouraging for an OP, because other stakeholders seldom seem to understand its value. However, these participants reported promoting early RTW via work modifications because they saw it as their central duty.

*Sometimes I’m overcome by a feeling that an OP is trying to achieve something that neither the patient nor the employer wants*.

*(Ida,* *group 4)*

*I agree. In those moments you ask yourself whether it makes any sense to try. You wonder whose affair this is anyway, mine or*?

*(Gary,* *group 4)*

*But we’ve been trained to do this… to support the remaining work ability and return to work, to avoid medication, and all kinds of things. And now we have these research results that when you are on a part-time sick leave you return to work earlier. And when you don’t stay away from work for many years you don’t end up in retirement, and so on. Somehow I have a feeling that employers pretty much regard this as a high-level rhetoric, and in practice it’s another thing. … We are trying to do something that would benefit the employer and the employee but neither is interested*.

*(Ida,* *group 4)*

## Discussion

### Main Findings and Their Interpretation

Five different areas of reasoning for recommending early RTW with work modifications were identified in focus groups discussions. For individual employees, OPs perceived early RTW both as a means of managing a medical condition and as a means of enhancing individuals’ well-being. For companies and work communities, OPs saw early RTW as producing financial benefits for companies and improving the well-being of supervisors and/or coworkers. For society, early RTW was seen to increase the work participation of all employees and to reduce societal costs related to work disability. Finally, for OPs themselves, early RTW was depicted to enhance their professional fulfillment by providing meaningful and satisfying duties.

Our results coincide with the justifications of early RTW after work injuries. MacEachen et al. [33] found four types of models of RTW. The physical model describes early RTW as a form of treatment or rehabilitation. The psychological model and the psychosocial model propose that being off work is associated with various negative consequences which may diminish the likelihood of RTW. The fiscal model focuses on societal level concerns and proposes that early RTW is a means of reducing public costs. In addition, our participants viewed encouraging early RTW as a meaningful task for themselves as OPs.

However, MacEachen et al. [33] argue that early RTW requires scientific scrutiny as it is often presented in policy and research as an unproblematic means to enhance work participation. Early RTW policies and guidelines in various jurisdictions are based on scientific evidence limited to certain conditions and sometimes even questionable logic about direction of causation. There is a lack of scientific knowledge on working conditions and individual health situations where early RTW is suited particularly well. Previous studies [7] have suggested that RTW may be more suitable in musculoskeletal disorders than in mental disorders.

The results of our study show that the interviewed Finnish OPs are familiar with the recommendations for early RTW, but these are not accepted without consideration to the RTW situation and context. OPs seem to acknowledge the various standpoints and perceptions of different stakeholders. Even if timely return to work was emphasized, and its positive effects were recognized for the workplace and the society, the OPs were concerned about possible harms of early RTW for employees. In addition, it was suggested that early RTW may be disadvantageous in some types of workplaces. All participants were not convinced about the evidence that early RTW will reduce company costs. When discussing the societal rationale of using early RTW, participants were concerned about the fairness of this practice. Our results suggest that practitioners’ experiences and perceptions should be consulted when guidelines and policies for their work are formulated.

From the point of view of training, we found especially interesting the topics which aroused negotiations between participants. It was debated whether early RTW would be a proper treatment for most medical conditions, or should be considered as the first option for employees regardless of age or the type of work. These considerations were even more intriguing, when the OPs recognized their own professional fulfillment as one of the rationales around RTW.

Our results suggest that there may be a lack of knowledge among OPs with regard to the usefulness of early RTW with work modifications in different medical conditions and various types of work and workplaces. More education may be needed to strengthen OPs’ confidence about when and how to use early RTW safely.

However, OPs’ negotiations about the application of early RTW may also be interpreted as unwillingness to expand OP role from handling mostly employees with prolonged sick leaves to proactively encouraging for early RTW. Effective forms of education should be applied to facilitate the adoption of this practice into OPS’ daily work. According to previous studies [34–37] adoption of new practices among health care practitioners is enhanced when educational interventions are based on learning methods that encourage participants’ reflection on their present knowledge and practices.

In addition to factors related to reasoning (knowledge and understanding), the practitioners’ adoption of practices may be influenced by factors related to e.g., environmental contexts and resources, social influences, and practitioners’ skills and conceptions of their professional roles [13–15, 38–40]. For our intervention, we will look next at these barriers and facilitators of early RTW among the participants.

### Strengths and Limitations of the Study

Our study moved beyond describing the experiences of practitioners with regard to (early) RTW to investigating their reasoning about this relatively new concept and practice in workplaces in Finland. We also used focus group data in a more versatile way than in most previous studies. We looked at the forms of discussion, i.e., the negotiations within groups, instead of analyzing the content of accounts only.

Qualitative research is evaluated by judging its conceptual generalizability, transferability, and systematic and rigorous application of data gathering and analysis methods [27–30]. Conceptual generalizability is generated through the identification of key elements in data that identify ways of thinking or ‘making sense’ of situations. This type of generalizability does not relate to representativeness of specific findings, but rather to the broader applicability of the concepts (the notion that physicians have varied reasons for supporting and not supporting early RTW that fall across the domains identified here). Our results describe qualitatively different rationales for using early RTW with work modifications and reservations for application under each rationale.

Transferability addresses the extent to which findings are transferable to other settings. Our sample size was small but included a variety of Finnish OPs. We propose that the types of reasoning identified in our analysis may be found more widely among Finnish OPs although new types of rationales may also be identified in future studies. In order to investigate whether the identified rationales for using early RTW are common among Finnish OPs, a survey might be needed in the future.

To ensure systematic and rigorous application of data gathering and analysis methods, data generation from the focus groups was planned carefully in order to answer the research questions. The principles and proceeding of the analysis are described in detail. Extracts of the data are presented to demonstrate how the data were interpreted. The extracts were translated from Finnish to English, and the accuracy of the translations was approved collectively.

We consider our analysis to be adequate, because it provides new knowledge, and includes relevant information about interaction between the data and the context [28]. In addition, our way of investigating reasoning and the related results may also inform the planning of educational activities more generally.

This study has some potential limitations. A critical question concerning validity is whether it is feasible to capture OPs’ reasoning about encouraging early RTW in focus groups discussions. Focus group discussions are a good method for gathering data on issues that are relevant to the participants [24–27]. However, focus group discussions are also affected by social contexts, e.g. by relationships between the participants. The degree of prior acquaintance and status relations among the participants may affect whether they choose to share all relevant thoughts, or whether they prefer to express opinions that they have learned earlier to be ‘acceptable’, and they think others want to hear or value especially. Participants may prefer conforming with previously expressed opinions to openly disagreeing with others. Whether they are supposed to interact with each other after the discussion, may affect their choices of conduct during the discussion.

We suggest, however, that these concerns are minor in the present study. Participants were of similar social and professional status within the groups. Some OPs had been workmates for a few months, and others were familiar to each other through having participated in the same educational events. The topic of the discussions was not especially socially sensitive for the participants, thus strong needs to monitor one’s speech were likely not present. These discussions might have as well occurred in the workplaces between colleagues. Expression of different point of views was actively encouraged.

## Conclusions

Encouraging early RTW with work modifications was perceived by OPs as a meaningful task and, to a large extent, beneficial for employees and several stakeholders. However, this practice was not accepted without consideration to the RTW situation and context. To enhance successful and timely RTW, OPs should be offered education that addresses their views regarding this practice.

## References

[1] van Duijn M, Eijkemans MJ, Koes BW, Koopmanschap MA, Burton KA, Burdorf A. The effects of timing on the cost-effectiveness of interventions for workers on sick leave due to low back pain. J Occup Environ Med. 2010;67: 744–750. 10.1136/oem.2009.049874 20833757

[2] Anema JR, Steenstra IA, Bongers PM, de Vet HC, Knol DL, Loisel P, et al Multidisciplinary rehabilitation for sub-acute low back pain: graded activity or workplace intervention or both? A randomized controlled trial. Spine 2007;32: 291–298. 17268258 10.1097/01.brs.0000253604.90039.ad

[3] van Oostrom SH, Driessen MT, de Vet HC, Franche RL, Schonstein E, Loisel P, et al Workplace interventions for preventing work disability. Cochrane Database Syst Rev. 2009; CD006955 10.1002/14651858.CD006955.pub2 19370664

[4] Martimo KP, Shiri R, Miranda H, Ketola R, Varonen H, Viikari-Juntura E. Effectiveness of an ergonomic intervention on the productivity of workers with upper-extremity disorders − a randomized controlled trial. Scand J Work Environ Health 2010;36: 25–33. 19960145 10.5271/sjweh.2880

[5] Shiri R, Martimo KP, Miranda H, Ketola R, Kaila-Kangas L, Liira H, et al The effect of workplace intervention on pain and sickness absence caused by upper-extremity musculoskeletal disorders. Scand J Work Environ Health 2011;37: 120–128. 10.5271/sjweh.3141 21218270

[6] van Vilsteren M, van Oostrom SH, de Vet HC, Franche RL, Boot CR, Anema JR. Workplace interventions to prevent work disability in workers on sick leave. Cochrane Database Syst Rev. 2015 10 5;10:CD006955 10.1002/14651858.CD006955.pub3 Review. 26436959 PMC9297123

[7] Nieuwenhuijsen K, Faber B, Verbeek JH, Neumeyer-Gromen A, Hees HL, Verhoeven AC, et al Interventions to improve return to work in depressed people. Cochrane Database Syst Rev. 2014; CD006237 10.1002/14651858.CD006237.pub3 33052607 PMC8094165

[8] Foss L, Gravseth HM, Kristensen P, Claussen B, Sivesind Mehlum I, Skyberk K. "Inclusive working life in Norway": a registry-based five-year follow-up study. J Occup Med Toxicol 2013; 8:19 10.1186/1745-6673-8-19 23829467 PMC3706356

[9] Gabbay M, Shiels C, Hillage J. Factors associated with the length of fit note-certified sickness episodes in the UK. Occup Environ Med. 2015; 72: 467–475. 10.1136/oemed-2014-102307 25713158

[10] Scheel IB, Hagen KB, Oxman AD. Active sick leave for patients with back pain. All the players onside, but still no action. Spine 2002;27: 654–659. 11884914 10.1097/00007632-200203150-00016

[11] Wainwright E, Wainwright D, Keogh E, Eccleston C. Fit for purpose? Using the fit note with patients with chronic pain: a qualitative study. Br J Gen Pract. 2011;61: e794–800. 10.3399/bjgp11X613133 22137416 PMC3223777

[12] Haukka E, Martimo K-P, Kivekäs T, Horppu R, Lallukka T, Solovieva S, et al Efficacy of temporary work modifications on disability related to musculoskeletal pain or depressive symptoms—study protocol for a controlled trial. BMJ Open 2015:5:e008300 10.1136/bmjopen-2015-008300 25986643 PMC4442237

[13] Grol R, Grimshaw J. From best evidence to best practice: effective implementation of change in patients' care. Lancet 2003;362: 1225–1230. 14568747 10.1016/S0140-6736(03)14546-1

[14] Michie S, Johnston M, Abraham C, Lawton R, Parker D & Walker A, on behalf of the "Psychological Theory" Group. Making psychological theory useful for implementing evidence based practice: a consensus approach. Qual Saf Health Care 2005;14: 26–33. 15692000 10.1136/qshc.2004.011155 PMC1743963

[15] Cane J, O’Connor D, Michie S. Validation of the theoretical domains framework for use in behaviour change and implementation research. Implementation Sci 2012;7: 37. 10.1186/1748-5908-7-37 PMC3483008 22530986

[16] Côté P, Clarke J, Deguire S, Frank JW, Yassi A. Chiropractors and return-to-work: The experiences of three Canadian focus groups. J Manipulative Physiol Ther. 2001:24: 309–316. 11416820 10.1067/mmt.2001.115267

[17] van Duijn M, Miedema H, Elders L, Burdorf A. Barriers for early return-to-work of workers with musculoskeletal disorders according to occupational health physicians and human resource managers. J Occup Rehabil. 2004;14: 31–41. 15055502 10.1023/b:joor.0000015009.00933.16

[18] Stigmar K, Grahn B, Ekdahl C. Work ability—experiences and perceptions among physicians. Disabil Rehabil. 2010;32: 1780–1789. 10.3109/09638281003678309 20230251

[19] Soklaridis S, Ammendolia C, Cassidy D. Looking upstream to understand low back pain and return to work. Psychosocial factors as the product of system issues. Soc Sci Med. 2010;71: 1557–1566. 10.1016/j.socscimed.2010.08.017 20864239

[20] Tiedtke C, Donceel P, Knops L, Désiron H, Dierckx de Casterle B, et al Supporting return-to-work in the face of legislation: Stakeholders’ experiences with return-to-work after breast cancer in Belgium. J Occup Rehabil. 2012; 22: 241–251. 10.1007/s10926-011-9342-0 22105670

[21] de Vries G, Koeter MW, Nabitz U, Hees HL, Schene AH. Return to work after sick leave due to depression: A conceptual analysis based on perspectives of patients, supervisors and occupational physicians. J Affect Disord. 2012;136: 1017–1026. 10.1016/j.jad.2011.06.035 21774988

[22] Hoefsmit N, Houkes I, Nijhuis FJN. Environmental and personal factors that support early return-to-work: A qualitative study using the ICF as a framework. Work 2014;48: 203–215. 10.3233/WOR-131657 23803441

[23] Wynne-Jones G, van der Windt D, Ong BN, Bishop A, Cowen J, Artus M, et al Perceptions of health professionals towards the management of back pain in the context of work: a qualitative study. BMC Musculoskelet Disord. 2014:15: 210 10.1186/1471-2474-15-210 24941952 PMC4073509

[24] Kitzinger J. Introducing focus groups. BMJ 1995;311: 299–302. 7633241 10.1136/bmj.311.7000.299 PMC2550365

[25] Hollander JA. The social contexts of focus groups. Journal of Contemporary Ethnography 2004;33: 602–637.

[26] Barbour RS. Making sense of focus groups. Med Educ. 2005;39: 742–750. 15960795 10.1111/j.1365-2929.2005.02200.x

[27] Barbour RS. Doing focus groups. Los Angeles: Sage; 2007.

[28] Mason J. Qualitative researching. London: Sage; 1996.

[29] Silverman D. Interpreting qualitative data Methods for analyzing talk, text and interaction. 2nd ed London: Sage; 2001.

[30] Green J, Thorogood N. Qualitative methods for health research. Thousand Oaks: Sage; 2014.

[31] Hsieh HF, Shannon SE. Three approaches to qualitative content analysis. Qual Health Res. 2005;15: 1277–1288. 16204405 10.1177/1049732305276687

[32] Elo S, Kyngäs H. The qualitative content analysis process. J Adv Nurs. 2007;62: 107–115. 10.1111/j.1365-2648.2007.04569.x 18352969

[33] MacEachen E, Ferrier S, Kosny A, Chambers L. A deliberation on 'hurt versus harm' logic in early-return-to-work policy. Policy Pract Health Saf. 2007;5: 41–62.

[34] Schaafsma F, Hugenholtz N, de Boer A, Smits P, Hulshof C, van Dijk F. Enhancing evidence-based advice of occupational physicians. Scand J Work Environ Health 2007;33: 368–378. 17973063 10.5271/sjweh.1156

[35] Smits PB, de Buisonjé CD, Verbeek JH, van Dijk FJ, Metz JC, ten Cate OJ. Problem-based learning versus lecture-based learning in postgraduate medical education. Scand J Work Environ Health 2003;29: 280–287. 12934721 10.5271/sjweh.732

[36] Shirazi M, Lonka K, Parikh SV, Ristner G, Alaeddini F, Sadeghi M, et al A tailored educational intervention improves doctor's performance in managing depression: a randomized controlled trial. J Eval Clin Pract. 2013;19: 16–24. 10.1111/j.1365-2753.2011.01761.x 21883718

[37] Mann KV. Continuing medical education In: Norman GR, van der Vleuten CPM, Newble DI, editors. International handbook of research in medical education. Dordrecht: Kluwer Academic Publishers; 2002 pp. 415–457.

[38] Fassier JB, Durand MJ, Loisel P. 2nd place, PREMUS best paper competition: implementing return-to-work interventions for workers with low-back pain—a conceptual framework to identify barriers and facilitators. Scand J Work Environ Health 2011;37: 99–108. 10.5271/sjweh.3138 21170504

[39] Gardner BT, Pransky G, Shaw WS, Hong QN, Loisel P. Researcher perspectives on competencies of return-to-work coordinators. Disabil Rehabil 2010;32: 72–78. 10.3109/09638280903195278 19925279

[40] Rebergen DS, Bruinvels DJ, Bos CM, van der Beek AJ, van Mechelen W. Return to work and occupational physicians' management of common mental health problems − process evaluation of a randomized controlled trial. Scand J Work Environ Health 2010;36: 488–498. 20798909 10.5271/sjweh.3084

